# Mental and Physical Well-Being Has Continued to Decline Among Patients Undergoing Orthopaedic Surgery from 2019 Through 2024: The Experience of a Single Specialized Academic Center in New York City

**DOI:** 10.2106/JBJS.OA.25.00353

**Published:** 2026-02-17

**Authors:** Zachary A. Trotzky, Ranqing Lan, Olivia M. Jochl, Patricia Friedmann, Mark A. Fontana, Catherine H. MacLean, Ernest L. Sink

**Affiliations:** 1 Hospital for Special Surgery, New York, New York; 2 Albert Einstein College of Medicine, Bronx, New York; 3 Healthcare Value Solutions, Ketchum, Idaho

## Abstract

**Background:**

A strong association exists between outcomes of orthopaedic surgery and patients' preoperative mental health. Therefore, population-wide trends and changes in well-being are of great interest to providers. We sought to describe the well-being among patients undergoing orthopaedic surgery before, during, and after the pandemic.

**Methods:**

Patients who underwent an orthopaedic surgical procedure at 1 specialty hospital in New York City between 2019 and August 2024 were split into prepandemic, pandemic, and postpandemic groups (n = 129,677). Preoperative Patient-Reported Outcome Measurement Information System Scale v1.2-Global Health (PROMIS-GH) mental health (MH) and physical health (PH) scores were analyzed in addition to the proportion of patients with low MH and PH. Demographic and clinical factors associated with low MH/PH were identified.

**Results:**

Median PROMIS MH and PH scores declined in the pandemic cohort and remained decreased in the postpandemic cohort. Compared with the prepandemic cohort, the proportion of patients with low MH increased in the pandemic cohort (11% vs. 5%) and increased further in the postpandemic cohort (14%). Compared with the prepandemic cohort, the proportion of patients with low PH increased in the pandemic cohort (37% vs. 25%) and increased further in the postpandemic cohort (40%). Relative to the prepandemic period, the odds of low MH adjusted for demographic, clinical, and social factors were 2.70 for the pandemic period and 3.46 for the postpandemic period; for low PH 1.95 and 2.21, respectively. Factors associated with low MH/PH included preoperative comorbidities, age, sex, ethnicity, insurance type, socioeconomic status, and surgery within the spine service.

**Conclusion:**

Our results suggest that mental and physical health among patients undergoing orthopaedic surgery in New York City worsened during the pandemic. In the postpandemic period, mental and physical health has continued to decline with a higher proportion of low MH and low PH compared with prepandemic and pandemic levels. The persistent decline observed past the pandemic period indicates that long-term social and societal factors may be associated with population-wide changes in psychosocial functioning rather than exclusively pandemic-specific determinants. In response to this negative trend, surgeons and institutions should consider additional preoperative supports for patients with diminished well-being.

**Level of Evidence:**

Level III—Retrospective cohort study. See Instructions for Authors for a complete description of levels of evidence.

## Introduction

The past 5 years including the COVID-19 pandemic and postpandemic periods have seen notable rises in stress, anxiety, and depression, altering the physical and mental needs of patient populations. Currently, more than 90% of United States (US) adults believe there is a mental health crisis in the US today^1^. Furthermore, more than half of adults reported they or a family member have experienced a severe mental health emergency^1^. Research about the psychosocial effects of COVID-19 has reported increases in anxiety, depression, sleep disorders, and substance abuse along with general mental health deterioration and emotional distress during periods of lockdown^2-8^. Disproportionate increases were observed in female patients, younger and middle-aged adults, racial/ethnic minority groups, those with fewer economic resources, and those residing in regions highly impacted by COVID-19^9-15^. Outside of pandemic-related determinants, numerous long-term social and societal factors, including economic instability, reduced social support, and country-level disparities in education and access to health resources, may be associated with continuous decreases in well-being. As such, while population-wide mental health has improved compared with the acute pandemic period, it has not fully returned to prepandemic levels^16,17^.

A strong association exists between outcomes of orthopaedic surgery and patients' preoperative mental health^18^. Prior studies have shown preoperative emotional distress to be predictive of worsened postoperative pain, function, and satisfaction following many common orthopaedic procedures^19-26^. Therefore, changes in well-being during and after the pandemic are of great interest to providers. The available literature describing health-related quality of life in orthopaedic surgery populations has compared prepandemic with pandemic periods, showing no meaningful changes in patient-reported physical and mental health^27,28^. However, to our knowledge, no studies have examined postpandemic well-being to determine whether mental health trends among the general population are consistent with patients undergoing orthopaedic surgery. Specific investigation of the postpandemic period may reveal whether population-wide changes in psychosocial functioning exist without pandemic-related exacerbation.

We evaluated whether there were changes in well-being among patients undergoing orthopaedic surgery in New York City before, during, and after the COVID-19 pandemic using self-reported mental and physical health as measured by the Patient-Reported Outcome Measurement Information System Scale v1.2-Global Health (PROMIS-GH). It is hypothesized that scores will continue to decline through the postpandemic period, even in the absence of pandemic-related health and safety protocols. Secondary aims included identifying demographic and clinical factors associated with “low” mental and physical health.

## Methods

### Study Design and Setting

This retrospective, hospital-wide, observational study was performed at an urban, tertiary academic orthopaedic center in New York City.

### Study Population

The study period was January 1, 2019, to August 19, 2024. During this time, 179,493 adults underwent an orthopaedic surgical procedure at the study site. Among these, 49,816 patients with incomplete or missing PROMIS-GH responses were excluded (Appendix 1), leaving 129,677 patients (72%) for analysis (Appendix 2). Included patients were stratified based on procedure date into the prepandemic, pandemic, or postpandemic cohorts. The start of the pandemic period was defined as March 11th, 2020, the date on which the World Health Organization formally declared the pandemic. The end of the pandemic period was defined as February 12th, 2023, the date on which mask mandates for healthcare facilities in New York City were lifted.

### Data and Endpoints

The PROMIS-GH (v1.2) is a validated, 10-item, self-reported measure, providing a mental health (MH) and physical health (PH) score. This instrument has been regularly administered at the study site within 30 days preoperatively for patients undergoing orthopaedic surgery since 2019^27-29^. Raw scores for MH and PH can be converted from PROMIS global scales into T-score values, standardized such that a score of 50 represents the average for the US general population^30^. Higher scores indicate better mental and physical health. The meaningful change threshold to make between-group comparisons ranges from 2 to 6 T-score points, with PROMIS leadership suggesting a threshold of 3 T-score points may be reasonable for most contexts^31,32^. We defined low MH as a T-score < 40 and low PH as a T-score < 42 (Appendix 1)^29,33-35^. See Appendix 1 for other included variables^36-39^.

### Statistical Analysis

One-way analysis of variance or the Kruskal-Wallis test, depending on normality, was used to compare continuous variables across the prepandemic, pandemic, and postpandemic groups. Chi-squared tests or Fisher exact tests, as appropriate, were used to compare categorical variables across the 3 groups. Mean PROMIS-GH scores and number of observations were plotted by quarter to observe trends over the study period. Unadjusted logistic regression was performed to determine the association between the pandemic and postpandemic periods with low MH and low PH compared with the prepandemic cohort. Adjusted multivariable logistic regressions were performed to assess the association between pandemic and postpandemic periods and low MH and PH controlling for demographic, underlying health risk, and social factors. Adjusted generalized variance inflation factor (GVIF) was calculated for each independent variable to detect multicollinearity (when 2 independent variables are highly correlated). Statistical analysis was performed by a biostatistician using R version 4.5.1.

## Results

### Patients

Each group was composed mostly of patients who live in New York State and identify as White and not Hispanic or Latino (Table I). Patients most commonly underwent surgery in the Adult Reconstruction & Joint Replacement Service, had commercial insurance, and had a low American Society of Anesthesiologists (ASA) status, Charlson Comorbidity Index (CCI), Area Deprivation Index (ADI), and Social Vulnerability Index.

**TABLE I:** Demographic Characteristics of Prepandemic, Pandemic, and Postpandemic Cohorts

Variable	Prepandemic (n = 25,817)		Pandemic (n = 65,612)		Postpandemic (n = 38,248)		p
	n	Mean ± SD	n	Mean ± SD	n	Mean ± SD	
Age at surgery (years)	25,817	55.8 ± 16.0	65,612	56.4 ± 16.1	38,248	57.2 ± 16.4	<0.001
BMI (kg/m^2^)	25,676	28.3 ± 5.8	65,312	28.3 ± 5.8	38,004	28.3 ± 5.8	0.41
ADI national percentile	24,373	13.8 ± 14.9	62,249	13.7 ± 14.7	35,762	13.8 ± 14.9	0.95
SVI overall percentile	25,757	39.7 ± 26.6	65,489	39.6 ± 26.5	38,173	39.7 ± 26.5	0.56

Variable	n	n%	n	n%	n	n%	p
ASA level							
1-2	22,701	89	57,336	88	32,942	87	0.001
3-5	2,850	11	7,785	12	5,009	13	
NA	266		491		297		
State of residence							<0.001
NY	17,181	67	44,543	68	25,763	67	
Other	8,630	33	21,056	32	12,481	33	
NA	6		13		4		
Sex							0.001
Male	12,791	50	32,869	50	18,692	49	
Female	13,026	50	32,743	50	19,556	51	
Race							<0.001
White	21,381	85	53,851	84	30,524	82	
Asian	970	4	2,589	4	1,848	5	
Black or African American	1,556	6	3,910	6	2,418	7	
Other	1,322	5	3,605	6	2,335	6	
NA	588		1,657		1,123		
Ethnicity							<0.001
Not Hispanic or Latino	23,589	93	59,480	93	34,352	92	
Hispanic or Latino	1,648	7	4,571	7	2,827	8	
NA	580		1,561		1,069		
CCI category							0.01
None	19,046	74	48,765	74	28,005	73	
Mild	6,027	23	14,942	23	9,092	24	
Moderate	576	2	1,488	2	920	2	
Severe	168	1	413	1	231	1	
NA	0		4		0		
BMI category							0.40
Normal	7,651	30	19,281	30	11,127	29	
Underweight	262	1	759	1	404	1	
Overweight	9,057	35	23,041	35	13,459	36	
Obese	8,585	34	21,934	34	12,829	34	
NA	262		597		429		
Age category							<0.001
18-29	2,442	9	5,968	9	3,423	9	
30-39	2,309	9	5,363	8	3,163	8	
40-49	3,020	12	7,240	11	3,845	10	
50-64	9,640	37	24,228	37	13,310	35	
65-79	7,484	29	20,243	31	12,761	33	
80+	922	4	2,570	4	1,746	5	
Primary service							<0.001
Adult reconstruction and joint replacement	9,212	36	25,043	38	15,903	42	
Foot and ankle	2,240	9	4,452	7	1,689	4	
Hand and upper extremity	2,134	8	4,775	7	2,386	6	
Limb lengthening	370	1	966	1	862	2	
Spine	3,167	12	8,330	13	5,197	14	
Sports medicine and shoulder	7,953	31	20,327	31	11,276	30	
Trauma	589	2	1,326	2	669	2	
NA	152		393		266		
Surgery							<0.001
Ambulatory	13,755	53	41,335	63	28,388	74	
Inpatient	12,062	47	24,277	37	9,860	26	
Insurance							<0.001
Commercial	17,411	68	43,773	67	25,004	66	
Medicaid	332	1	741	1	419	1	
Medicare	6,944	27	18,620	29	11,633	31	
Worker's comp and no fault	892	3	2,067	3	842	2	
NA	238		411		350		

ADI = Area Deprivation Index, ASA = American Society of Anesthesiologists, BMI = Body Mass Index, CCI = Charlson Comorbidity Index, SVI = Social Vulnerability Index, NY = New York, and NA = not available.

### Change in PROMIS-GH Scores

**Fig. 1:**
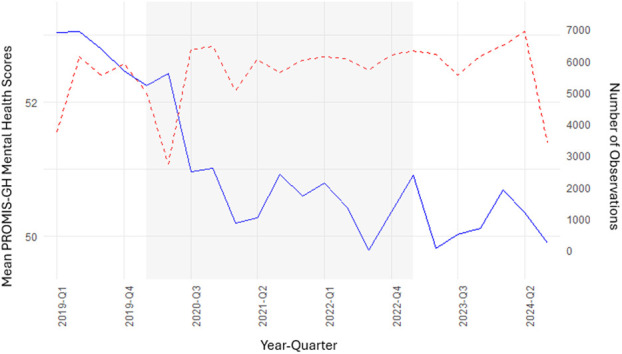

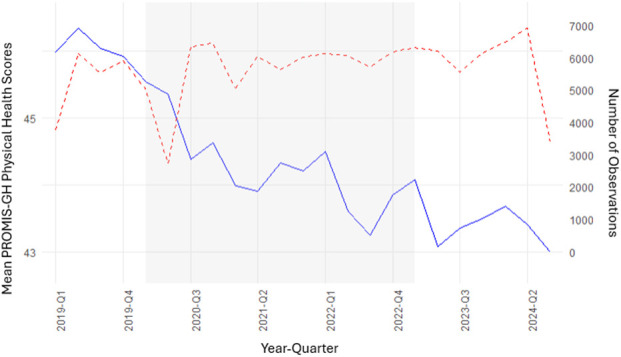
**Figs 1-A and 1-B** Mean PROMIS-GH mental health and physical health scores are plotted by quarter. Mean scores are shown on the blue line. The number of observations is shown on the dotted red line. The shaded area indicates the pandemic period. PROMIS-GH = Patient-Reported Outcome Measurement Information System Scale v1.2-Global Health.

Compared with the prepandemic cohort, mean preoperative PROMIS MH and PH scores declined in the pandemic cohort and remained decreased in the postpandemic cohort (Figs 1-A and 1-B). The change in median pandemic and postpandemic MH scores compared with prepandemic exceeded the lower bound of the defined 2- to 6-point meaningful change threshold range, while 25th percentile scores were >3 points less (Table II). Compared with the prepandemic cohort, the proportion of patients with low MH increased in the pandemic cohort (11% vs. 5%) and increased further in the postpandemic cohort (14%). The increased likelihood of having low MH in the pandemic and postpandemic cohorts corresponded to odds ratios (ORs) of 2.57 (95% confidence interval [CI] 2.40-2.75; p < 0.001) and 3.29 (95% CI 3.07-3.52; p < 0.001), respectively, according to unadjusted regression models. The change in median postpandemic PH scores compared with prepandemic exceeded the lower bound of the defined 2- to 6-point meaningful change threshold, while pandemic and postpandemic 25th percentile scores were >3 points less. Compared with the prepandemic cohort, the proportion of patients with low PH increased in the pandemic cohort (37% vs. 25%) and increased further in the postpandemic cohort (40%). The increased likelihood of having low PH in the pandemic and postpandemic cohorts, relative to the prepandemic cohort, corresponded to ORs of 1.78 (95% CI 1.72-1.84; p < 0.001) and 2.03 (95% CI 1.96-2.11; p < 0.001), respectively, according to unadjusted regression models.

**TABLE II:** PROMIS-GH Scores Across Prepandemic, Pandemic, and Postpandemic Cohorts

Sample Characteristics	Prepandemic Cohort		Pandemic Cohort		Postpandemic Cohort		Significance
	Median (IQR)	Range	Median (IQR)	Range	Median (IQR)	Range	p
Preop PROMIS MH	53.3 (7.7)	21.2-67.6	50.8 (12.5)	16.0-67.6	50.8 (12.5)	21.2-67.6	<0.001
25th percentile	48.3		43.5		43.5		
75th percentile	56.0		56.0		56.0		
Preop PROMIS PH	44.9 (8.5)	16.2-67.7	44.0 (13.4)	15.0-67.7	42.3 (13.4)	16.2-67.7	<0.001
25th percentile	42.3		37.4		37.4		
75th percentile	50.8		50.8		50.8		

Sample Characteristics	n	Proportion of Prepandemic Cohort %	n	Proportion of Pandemic Cohort %	n	Proportion of Postpandemic Cohort %	p
Patients with low mental health (<40)	1,218	5	7,407	11	5,327	14	<0.001
Patients with low physical health (<42)	6,448	25	24,514	37	15,437	40	<0.001

IQR = interquartile range, MH = mental health, PH = physical health, and PROMIS-GH = Patient-Reported Outcome Measurement Information System Scale v1.2-Global Health.

### Factors Associated with Low MH and Low PH

When controlling for demographic, clinical, and social covariates, being in the pandemic and postpandemic groups was associated with further increased likelihoods of having low MH and low PH compared with the unadjusted models (Table III). Other factors associated with low MH and low PH included an increased ASA and CCI, being middle-aged (ages 40-49, 50-64), abnormal Body Mass Index (BMI), identifying as female or Hispanic or Latino, having surgery within the spine service, having public or employer-mandated/liability insurance, and an increased ADI. Identifying as a racial minority was associated with low PH but not low MH. Ambulatory (versus inpatient) surgery was associated with a reduced likelihood of having low MH or low PH. For all independent variables, adjusted GVIF was ≤1.25 in each model, suggesting no evidence of multicollinearity.

**TABLE III:** Factors Associated with Low Mental Health and Low Physical Health

	Mental Health			Physical Health		
Factor	aOR Estimate	95% CI	p	aOR Estimate	95% CI	p
Intercept	0.03	0.02-0.03	<0.001	0.10	0.10-0.11	<0.001
Group by date of surgery						
Prepandemic group (reference)	1.00			1.00		
Pandemic group	**2.70**	**2.52-2.89**	**<0.001**	**1.95**	**1.88-2.02**	**<0.001**
Postpandemic group	**3.46**	**3.22-3.72**	**<0.001**	**2.21**	**2.12-2.30**	**<0.001**
Setting						
Inpatient surgery (reference)	1.00			1.00		
Ambulatory surgery	**0.79**	**0.75-0.83**	**<0.001**	**0.80**	**0.77-0.82**	**<0.001**
ASA level						
1-2 (reference)	1.00			1.00		
3-5	**1.41**	**1.33-1.49**	**<0.001**	**1.59**	**1.52-1.66**	**<0.001**
Age group						
18-29 (reference)	1.00			1.00		
30-39	1.12	0.99-1.26	0.07	**1.45**	**1.34-1.56**	**<0.001**
40-49	**1.31**	**1.17-1.47**	**<0.001**	**1.65**	**1.54-1.77**	**<0.001**
50-64	**1.31**	**1.19-1.45**	**<0.001**	**1.72**	**1.61-1.83**	**<0.001**
65-79	0.98	0.87-1.10	0.72	**1.37**	**1.27-1.47**	**<0.001**
80+	1.11	0.96-1.27	0.16	**1.62**	**1.47-1.78**	**<0.001**
BMI category						
Normal (reference)	1.00			1.00		
Underweight	**1.32**	**1.10-1.58**	**0.002**	**1.15**	**1.01-1.31**	**0.03**
Overweight	**1.08**	**1.02-1.14**	**0.01**	**1.25**	**1.20-1.29**	**<0.001**
Obese	**1.24**	**1.18-1.31**	**<0.001**	**1.81**	**1.75-1.88**	**<0.001**
CCI category						
None (reference)	1.00			1.00		
Mild	**1.27**	**1.21-1.33**	**<0.001**	**1.42**	**1.38-1.47**	**<0.001**
Moderate	**1.50**	**1.35-1.67**	**<0.001**	**1.88**	**1.72-2.05**	**<0.001**
Severe	**1.47**	**1.21-1.77**	**<0.001**	**1.89**	**1.60-2.23**	**<0.001**
Sex						
Male (reference)	1.00			1.00		
Female	**1.36**	**1.30-1.41**	**<0.001**	**1.73**	**1.69-1.78**	**<0.001**
Race						
White (reference)	1.00			1.00		
Asian	1.04	0.94-1.16	0.41	**1.15**	**1.08-1.23**	**<0.001**
Black or African American	1.00	0.93-1.08	0.96	**1.06**	**1.00-1.13**	**0.03**
Other	1.03	0.94-1.13	0.54	**1.16**	**1.09-1.24**	**<0.001**
Ethnicity						
Not Hispanic or Latino (reference)	1.00			1.00		
Hispanic or Latino	**1.14**	**1.05-1.24**	**0.002**	**1.12**	**1.06-1.19**	**<0.001**
Primary service						
Adult reconstruction and joint replacement (reference)	1.00			1.00		
Foot and ankle	**0.52**	**0.47-0.58**	**<0.001**	**0.42**	**0.40-0.45**	**<0.001**
Hand and upper extremity	**0.59**	**0.53-0.66**	**<0.001**	**0.47**	**0.44-0.50**	**<0.001**
Limb lengthening	1.10	0.95-1.27	0.19	**0.80**	**0.72-0.89**	**<0.001**
Spine	**1.86**	**1.76-1.95**	**<0.001**	**1.69**	**1.62-1.76**	**<0.001**
Sports medicine and shoulder	**0.56**	**0.53-0.60**	**<0.001**	**0.54**	**0.52-0.56**	**<0.001**
Trauma	**0.65**	**0.54-0.76**	**<0.001**	**0.62**	**0.56-0.69**	**<0.001**
Insurance/payer types						
Commercial (reference)	1.00			1.00		
Medicaid	**1.61**	**1.37-1.89**	**<0.001**	**1.44**	**1.27-1.63**	**<0.001**
Medicare	**1.36**	**1.27-1.45**	**<0.001**	**1.28**	**1.22-1.34**	**<0.001**
Worker's comp and no fault	**1.46**	**1.31-1.63**	**<0.001**	**1.57**	**1.45-1.70**	**<0.001**
ADI national percentile	**1.01**	**1.00-1.01**	**<0.001**	**1.01**	**1.00-1.01**	**<0.001**
SVI overall percentile	**1.00**	**1.00-1.00**	**<0.001**	**1.00**	**1.00-1.00**	**<0.001**

ASA = American Society of Anesthesiologists, ADI = Area Deprivation Index, aOR = adjusted odds ratio, BMI = Body Mass Index, CCI = Charlson Comorbidity Index, and SVI = Social Vulnerability Index. Significant factors (p < .05) are highlighted in bold.

## Discussion

The regions most affected by COVID-19 were estimated to have the largest increases in anxiety and depression due to a combination of more stringent lockdowns, harsh economic effects, and exposure to a higher number of stressors^14,40^. Consistent with these reports, patients in the pandemic cohort reported lower median PROMIS MH and PH scores as well as a higher proportion of low MH and low PH than the prepandemic group. Median MH scores were >2 points lower, while 25th percentile MH and PH scores were >3 points lower, indicating a meaningful decrease in mental and physical health, particularly among patients in the lowest quartile of PROMIS scores. At the population level, these changes translated to substantial absolute numbers of individuals experiencing clinically significant worsening, evidenced by the increased proportion of low MH and PH. These findings contrast with previous studies assessing changes in PROMIS scores or measures of anxiety and depression at other US orthopaedic tertiary care locations including northeast Ohio, the California Bay Area, and an orthopaedic population based in St. Louis, Missouri, which found no meaningful change during the pandemic^27,28,41^. Therefore, providers in New York City and other communities most vulnerable to the pandemic should consider increased vigilance for a decline in health-related quality of life. Importantly, the experience of our single institution in New York City may not generalize to other cities, more rural areas, or the broader US. New York City experienced an early, intense surge of COVID-19 with seroprevalence and infection-fatality risk that exceeded national and international averages. The overwhelming burden of this rapid epidemic growth on New York City healthcare systems may distinguish its impact on overall health status from other areas of the country. Most notably in our study, patients in the postpandemic cohort reported decreased median PH and further increases in the proportion of low MH and PH, suggesting that patient health and functioning has continued to decline despite the conclusion of pandemic-related distancing, masking, and sanitization protocols. While the trajectory of PROMIS-GH scores reflects a pandemic-related exacerbation of worse perceived health, the decrease in the postpandemic period indicates social and societal factors may have been the primary drivers of a long-term continuous decline. These factors, due to a combination of ongoing secular trends, increased socioeconomic barriers, and evolving social dynamics, may have begun to negatively influence well-being before the onset of the pandemic.

As scores below our threshold values have been linked to worse clinically relevant health outcomes^33,34^, the PROMIS-GH can serve as a useful screening tool for identifying low MH and low PH patients who may benefit from deliberative and personalized preoperative management. In addition to routine screening, improvements in multidisciplinary care such as access to mental health providers, specific patient education, innovative physical therapy, and recommendations for relaxation techniques can help to optimize recovery^42-44^. Preoperative psychological therapy/mental health counseling and preoperative physical therapy (prehabilitation) have both demonstrated associations with improved postoperative pain, function, and quality of life and can form the basis for service-tailored programs^45,46^. Future research may evaluate whether the sustained decline in mental and physical health observed in this study is related to changes in postoperative outcomes compared with prepandemic periods.

The disproportionate effects of the COVID-19 pandemic across demographic groups have been well-documented^47^. Our results reveal contemporary health disparities for orthopaedic surgery, suggesting that various demographic and clinical factors remain associated with low MH and PH. Female, middle-aged, Hispanic or Latino, nonprivate insurance, comorbidities, abnormal BMI, and socioeconomic disadvantage have all demonstrated relationships with worsened mental and physical health during the pandemic^48-53^. Compared with other service lines, patients undergoing spine surgery were most likely to report low MH and PH. As spine surgery patients may present with more severe, widespread baseline pain and functional impairment, this group may have been more susceptible to deterioration with pandemic-related delays in care^54,55^. Identifying risk factors associated with decreased MH and PH helps to specify the subpopulations that warrant the most immediate response for addressing preoperative well-being. Even with the implementation of systematic preventative measures, each patient in the post-COVID era will present with a unique physical, social, and emotional profile. As low MH and PH become more pervasive, providers should remain cognizant of opportunities to support patients individually^56^.

This study has several limitations. First, the race and ethnicity distribution of the study sample is not representative of the overall US or the greater New York City population, which may limit generalizability. However, it is similar to patients hospitalized in the US for orthopaedic procedures as per the National Inpatient Sample^57^ and, thus, likely generalizable to the broader orthopaedic population. Selection bias may be present as a portion of patients were excluded due to missing PROMIS-GH scores. Nevertheless, the prepandemic, pandemic, and postpandemic groups were comparable in terms of nearly all demographic and clinical factors. Furthermore, the number of observations (PROMIS-GH scores) per quarter remained relatively constant. While statistical differences were observed across groups in terms of demographics and PROMIS-GH scores, interpretation of p-values should be more cautious in this cohort given the large sample size as even small absolute differences will reach “significance.” Therefore, we have interpreted changes in PROMIS-GH scores relative to the meaningful change threshold (2-6 T-score points) and established cutoffs for low PH and low MH to provide clinical relevance to magnitudes of change.

PROMIS-GH scores at a specialized academic medical center in New York City reveal worsening mental and physical health among patients undergoing orthopaedic surgery since the onset of COVID-19 that has continued to deteriorate following the pandemic’s conclusion. It is advised that additional, multidisciplinary preoperative measures be considered to mitigate population-wide trends in mental health. Future research using nationally representative cohorts may confirm these results or assess the relationship between postpandemic well-being and patient satisfaction.

## Appendix

Supporting material provided by the authors is posted with the online version of this article as a data supplement at jbjs.org (http://links.lww.com/JBJSOA/B115). This content was not copyedited or verified by JBJS.

## References

[1] Kaiser Family Foundation (KFF), CNN. KFF/CNN Mental Health in America Survey. Published October 5, 2022. https://www.kff.org/mental-health/report/kff-cnn-mental-health-in-america-survey/. Accessed June 17, 2025.

[2] The Lancet P. COVID-19 and mental health. Lancet Psychiatry. 2021;8(2):87. 33485416 10.1016/S2215-0366(21)00005-5 PMC7825966

[3] Bouza E Arango C Moreno C Gracia D Martín M Pérez V Lázaro L Ferre F Salazar G Tejerina-Picado F Navío M Granda Revilla J Palomo E Gil-Monte PR. Impact of the COVID-19 pandemic on the mental health of the general population and health care workers. Rev Esp Quimioter. 2023;36(2):125-43. 36800778 10.37201/req/018.2023 PMC10066913

[4] Zhang SX Chen RZ Xu W Yin A Dong RK Chen BZ Delios AY Miller S McIntyre RS Ye W Wan X. A systematic review and meta-analysis of symptoms of anxiety, depression, and insomnia in Spain in the COVID-19 crisis. Int J Environ Res Public Health. 2022;19(2):1018. 35055841 10.3390/ijerph19021018 PMC8775436

[5] Jones EAK Mitra AK Bhuiyan AR. Impact of COVID-19 on mental health in adolescents: a systematic review. Int J Environ Res Public Health. 2021;18(5):2470. 33802278 10.3390/ijerph18052470 PMC7967607

[6] Pierce M Hope H Ford T Hatch S Hotopf M John A Kontopantelis E Webb R Wessely S McManus S Abel KM. Mental health before and during the COVID-19 pandemic: a longitudinal probability sample survey of the UK population. Lancet Psychiatry. 2020;7(10):883-92. 32707037 10.1016/S2215-0366(20)30308-4 PMC7373389

[7] Prati G Mancini AD. The psychological impact of COVID-19 pandemic lockdowns: a review and meta-analysis of longitudinal studies and natural experiments. Psychol Med. 2021;51(2):201-11. 33436130 10.1017/S0033291721000015 PMC7844215

[8] Henssler J Stock F van Bohemen J Walter H Heinz A Brandt L. Mental health effects of infection containment strategies: quarantine and isolation-a systematic review and meta-analysis. Eur Arch Psychiatry Clin Neurosci. 2021;271(2):223-34. 33025099 10.1007/s00406-020-01196-x PMC7538183

[9] Fountoulakis KN Karakatsoulis G Abraham S Adorjan K Ahmed HU Alarcón RD Arai K Auwal SS Berk M Bjedov S Bobes J Bobes-Bascaran T Bourgin-Duchesnay J Bredicean CA Bukelskis L Burkadze A Abud IIC Castilla-Puentes R Cetkovich M Colon-Rivera H Corral R Cortez-Vergara C Crepin P De Berardis D Zamora Delgado S De Lucena D De Sousa A Stefano RD Dodd S Elek LP Elissa A Erdelyi-Hamza B Erzin G Etchevers MJ Falkai P Farcas A Fedotov I Filatova V Fountoulakis NK Frankova I Franza F Frias P Galako T Garay CJ Garcia-Álvarez L García-Portilla MP Gonda X Gondek TM González DM Gould H Grandinetti P Grau A Groudeva V Hagin M Harada T Hasan MT Hashim NA Hilbig J Hossain S Iakimova R Ibrahim M Iftene F Ignatenko Y Irarrazaval M Ismail Z Ismayilova J Jacobs A Jakovljević M Jakšić N Javed A Kafali HY Karia S Kazakova O Khalifa D Khaustova O Koh S Kopishinskaia S Kosenko K Koupidis SA Kovacs I Kulig B Lalljee A Liewig J Majid A Malashonkova E Malik K Malik NI Mammadzada G Mandalia B Marazziti D Marčinko D Martinez S Matiekus E Mejia G Memon RS Martínez XEM Mickevičiūtė D Milev R Mohammed M Molina-López A Morozov P Muhammad NS Mustač F Naor MS Nassieb A Navickas A Okasha T Pandova M Panfil AL Panteleeva L Papava I Patsali ME Pavlichenko A Pejuskovic B Pinto Da Costa M Popkov M Popovic D Raduan NJN Ramírez FV Rancans E Razali S Rebok F Rewekant A Flores ENR Rivera-Encinas MT Saiz P de Carmona MS Martínez DS Saw JA Saygili G Schneidereit P Shah B Shirasaka T Silagadze K Sitanggang S Skugarevsky O Spikina A Mahalingappa SS Stoyanova M Szczegielniak A Tamasan SC Tavormina G Tavormina MGM Theodorakis PN Tohen M Tsapakis EM Tukhvatullina D Ullah I Vaidya R Vega-Dienstmaier JM Vrublevska J Vukovic O Vysotska O Widiasih N Yashikhina A Prezerakos PE Smirnova D. Results of the COVID-19 mental health international for the general population (COMET-G) study. Eur Neuropsychopharmacol. 2022;54:21-40. 34758422 10.1016/j.euroneuro.2021.10.004 PMC8609892

[10] Lieneck C Bosworth M Weaver E Heinemann K Patel J. Protective and non-protective factors of mental health distress in the United States during the COVID-19 pandemic: a systematic review. Medicina (Kaunas). 2021;57(12):1377. 34946322 10.3390/medicina57121377 PMC8708293

[11] Collaborators C-MD. Global prevalence and burden of depressive and anxiety disorders in 204 countries and territories in 2020 due to the COVID-19 pandemic. Lancet. 2021;398(10312):1700-12. 34634250 10.1016/S0140-6736(21)02143-7 PMC8500697

[12] Cao W Fang Z Hou G Han M Xu X Dong J Zheng J. The psychological impact of the COVID-19 epidemic on college students in China. Psychiatry Res. 2020;287:112934. 32229390 10.1016/j.psychres.2020.112934 PMC7102633

[13] Czeisler M Lane RI Petrosky E Wiley JF Christensen A Njai R Weaver MD Robbins R Facer-Childs ER Barger LK Czeisler CA Howard ME Rajaratnam SM. Mental health, substance use, and suicidal ideation during the COVID-19 pandemic - united States, June 24-30, 2020. MMWR Morb Mortal Wkly Rep. 2020;69(32):1049-57. 32790653 10.15585/mmwr.mm6932a1 PMC7440121

[14] Cui J Lu J Weng Y Yi GY He W. COVID-19 impact on mental health. BMC Med Res Methodol. 2022;22(1):15. 35026998 10.1186/s12874-021-01411-w PMC8758244

[15] Magesh S John D Li WT Li Y Mattingly-app A Jain S Chang EY Ongkeko WM. Disparities in COVID-19 outcomes by race, ethnicity, and socioeconomic status: a systematic-review and meta-analysis. JAMA Netw Open. 2021;4(11):e2134147. 34762110 10.1001/jamanetworkopen.2021.34147 PMC8586903

[16] Mauz E Walther L Junker S Kersjes C Damerow S Eicher S Hölling H Müters S Peitz D Schnitzer S Thom J. Time trends in mental health indicators in Germany's adult population before and during the COVID-19 pandemic. Front Public Health. 2023;11:1065938. 36908429 10.3389/fpubh.2023.1065938 PMC9995751

[17] Islam A Mahbuba P Ahmed T Haque S. Modifiable and nonmodifiable factors associated with anxiety, depression, and stress after one year of the COVID-19 pandemic. PLoS One. 2023;18(3):e0283422. 36952537 10.1371/journal.pone.0283422 PMC10035880

[18] Graham LA Hawn MT Dasinger EA Baker SJ Oriel BS Wahl TS Richman JS Copeland LA Itani KM Burns EA Whittle J Morris MS. Psychosocial determinants of readmission after surgery. Med Care. 2021;59(10):864-71. 34149017 10.1097/MLR.0000000000001600 PMC8425630

[19] Sharma AK Elbuluk AM Gkiatas I Kim JM Sculco PK Vigdorchik JM. Mental health in patients undergoing orthopaedic surgery: diagnosis, management, and outcomes. JBJS Rev. 2021;9(7):e20.00169. 10.2106/JBJS.RVW.20.00169 34297704

[20] Ayers DC Franklin PD Ring DC. The role of emotional health in functional outcomes after orthopaedic surgery: extending the biopsychosocial model to orthopaedics: AOA critical issues. J Bone Joint Surg. 2013;95(21):e165. 24196477 10.2106/JBJS.L.00799 PMC3808180

[21] Trief PM Ploutz-Snyder R Fredrickson BE. Emotional health predicts pain and function after fusion: a prospective multicenter study. Spine (Phila Pa 1976). 2006;31(7):823-30. 16582857 10.1097/01.brs.0000206362.03950.5b

[22] Anakwe RE Jenkins PJ Moran M. Predicting dissatisfaction after total hip arthroplasty: a study of 850 patients. J Arthroplasty. 2011;26(2):209-13. 20462736 10.1016/j.arth.2010.03.013

[23] Heck DA Robinson RL Partridge CM Lubitz RM Freund DA. Patient outcomes after knee replacement. Clin Orthop Relat Res. 1998;356:93-110. 10.1097/00003086-199811000-00015 9917673

[24] Clay FJ Newstead SV McClure RJ. A systematic review of early prognostic factors for return to work following acute orthopaedic trauma. Injury. 2010;41(8):787-803. 20435304 10.1016/j.injury.2010.04.005

[25] Paredes AZ Hyer JM Diaz A Tsilimigras DI Pawlik TM. The impact of mental illness on postoperative outcomes among medicare beneficiaries: a missed opportunity to help surgical patients?. Ann Surg. 2020;272(3):419-25. 32568745 10.1097/SLA.0000000000004118

[26] Jochl OM Afetse EK Garg S Kanakamedala AC Lind DR Hinz M Rizzo M Millett PJ Ruzbarsky J Provencher MT. The impact of mental health conditions on clinical and functional outcomes after shoulder arthroplasty: a systematic review. JSES Rev Rep Tech. 2024;4(3):371-8. 39157244 10.1016/j.xrrt.2024.04.014 PMC11329040

[27] Hollenberg AM Yanik EL Hannon CP Calfee RP O'Keefe RJ. Did the physical and mental health of orthopaedic patients change after the onset of the COVID-19 pandemic? Clin Orthop Relat Res. 2023;481(5):935-44. 36696142 10.1097/CORR.0000000000002555 PMC10097584

[28] Lapin BR Tang WHW Honomichl R Hogue O Katzan IL. Evidence of stability in patient-reported global health during the COVID-19 pandemic. Value in Health. 2021;24(11):1578-85. 34711357 10.1016/j.jval.2021.06.009 PMC8325511

[29] Hays RD Spritzer KL Thompson WW Cella D. U.S. general population estimate for “Excellent” to “Poor” self-rated health item. J Gen Intern Med. 2015;30(10):1511-6. 25832617 10.1007/s11606-015-3290-x PMC4579204

[30] Katzan IL Lapin B PROMIS GH. Patient-reported outcomes measurement information system global health) scale in stroke: a validation study. Stroke. 2018;49(1):147-54. 29273595 10.1161/STROKEAHA.117.018766

[31] Meaningful Change for PROMIS. HealthMeasures. https://www.healthmeasures.net/score-and-interpret/interpret-scores/promis/meaningful-change. Accessed June 23, 2025.

[32] Terwee CB Peipert JD Chapman R Lai JS Terluin B Cella D Griffiths P Mokkink LB. Minimal important change (MIC): a conceptual clarification and systematic review of MIC estimates of PROMIS measures. Qual Life Res. 2021;30(10):2729-54. 34247326 10.1007/s11136-021-02925-y PMC8481206

[33] Blumenthal KJ Chang Y Ferris TG Spirt JC Vogeli C Wagle N Metlay JP. Using a self-reported global health measure to identify patients at high risk for future healthcare utilization. J Gen Intern Med. 2017;32(8):877-82. 28341894 10.1007/s11606-017-4041-y PMC5515787

[34] Nagaraja V Mara C Khanna PP Namas R Young A Fox DA Laing T McCune WJ Dodge C Rizzo D Almackenzie M Khanna D. Establishing clinical severity for PROMIS(®) measures in adult patients with rheumatic diseases. Qual Life Res. 2018;27(3):755-64. 28983738 10.1007/s11136-017-1709-z PMC5845827

[35] PROMIS Score Cut Points. HealthMeasures. https://www.healthmeasures.net/score-and-interpret/interpret-scores/promis/promis-score-cut-points. Accessed June 17, 2025.

[36] Rius C Pérez G Martínez JM Bares M Schiaffino A Gispert R Fernández E. An adaptation of Charlson comorbidity index predicted subsequent mortality in a health survey. J Clin Epidemiol. 2004;57(4):403-8. 15135843 10.1016/j.jclinepi.2003.09.016

[37] Huang YQ Gou R Diao YS Yin QH Fan WX Liang YP Chen Y Wu M Zang L Li L Zang J Cheng L Fu P Liu F. Charlson comorbidity index helps predict the risk of mortality for patients with type 2 diabetic nephropathy. J Zhejiang Univ Sci B. 2014;15(1):58-66. 24390745 10.1631/jzus.B1300109 PMC3891119

[38] Lupei MI Chipman JG Beilman GJ Oancea SC Konia MR. The association between ASA status and other risk stratification models on postoperative intensive care unit outcomes. Anesth Analgesia. 2014;118(5):989-94. 10.1213/ANE.0000000000000187 24781569

[39] Wolters U Wolf T Stützer H Schröder T. ASA classification and perioperative variables as predictors of postoperative outcome. Br J Anaesth. 1996;77(2):217-22. 8881629 10.1093/bja/77.2.217

[40] Ettman CK Abdalla SM Cohen GH Sampson L Vivier PM Galea S. Prevalence of depression symptoms in US adults before and during the COVID-19 pandemic. JAMA Netw Open. 2020;3(9):e2019686. 32876685 10.1001/jamanetworkopen.2020.19686 PMC7489837

[41] Ziadni MS You DS Cramer EM Anderson SR Hettie G Darnall BD Mackey SC. The impact of COVID-19 on patients with chronic pain seeking care at a tertiary pain clinic. Sci Rep. 2022;12(1):6435. 35440688 10.1038/s41598-022-10431-5 PMC9017421

[42] Davis MLDRG Brenner M Best Practices in the Management of Orthopaedic Trauma. American College of Surgeons; 2015.

[43] Szeverenyi C Kekecs Z Johnson A Elkins G Csernatony Z Varga K. The use of adjunct psychosocial interventions can decrease postoperative pain and improve the quality of clinical care in orthopedic surgery: a systematic review and meta-analysis of randomized controlled trials. The J Pain. 2018;19(11):1231-52. 29803669 10.1016/j.jpain.2018.05.006

[44] Han H Chen C Sheng R Wang S. Psychological intervention based on cognitive behavioral therapy for patients with orthopedic surgical anxiety. Medicine (Baltimore). 2024;103(35):e39401. 39213206 10.1097/MD.0000000000039401 PMC11365644

[45] Hall AE Nguyen NH Cascavita CT Shariati K Patel AK Chen W Kang Y Ren X Tseng CH Hidalgo MA Lee JC. The impact of psychological prehabilitation on surgical outcomes: a meta-analysis and meta-regression. Ann Surg. 2025;281(6):928-41. 39969855 10.1097/SLA.0000000000006677

[46] Punnoose A Claydon-Mueller LS Weiss O Zhang J Rushton A Khanduja V. Prehabilitation for patients undergoing orthopedic surgery: a systematic review and meta-analysis. JAMA Netw Open. 2023;6(4):e238050. 37052919 10.1001/jamanetworkopen.2023.8050 PMC10102876

[47] Bathina KC Ten Thij M Valdez D Rutter LA Bollen J. Declining well-being during the COVID-19 pandemic reveals US social inequities. PLoS One. 2021;16(7):e0254114. 34237087 10.1371/journal.pone.0254114 PMC8266050

[48] Zhu K Wang S Yue Y Smith BA Zhang ZF Freudenheim JL Niu Z Zhang J Smith E Ye J Cao Y Zhang J Hennessy DA Lei L Mu L. Disparities in insecurity, social support, and family relationships in association with poor mental health among US adults during the COVID-19 pandemic. Sci Rep. 2023;13(1):9731. 37322075 10.1038/s41598-023-35981-0 PMC10272217

[49] Chakrabarti S Hamlet LC Kaminsky J Subramanian SV. Association of human mobility restrictions and Race/ethnicity-based, sex-based, and income-based factors with inequities in well-being during the COVID-19 pandemic in the United States. JAMA Netw Open. 2021;4(4):e217373. 33825836 10.1001/jamanetworkopen.2021.7373 PMC8027913

[50] Luo Y Li Q Jeong H Cheatham L. The association between social determinants of health and psychological distress during the COVID-19 pandemic: a secondary analysis among four racial/ethnic groups. BMC Public Health. 2022;22(1):2193. 36443734 10.1186/s12889-022-14486-x PMC9702892

[51] Lee H Singh GK. Monthly trends in self-reported health status and depression by race/ethnicity and socioeconomic status during the COVID-19 pandemic, United States, April 2020 - May 2021. Ann Epidemiol. 2021;63:52-62. 34358622 10.1016/j.annepidem.2021.07.014 PMC8435379

[52] Centers for Disease Control and Prevention. Obesity RE, and COVID-19. CDC. Updated approximately 1.4 years ago. Available at: https://www.cdc.gov/obesity/data/obesity-and-covid-19.html. Accessed June 17, 2025.

[53] Maehl N Bleckwenn M Riedel-Heller SG Mehlhorn S Lippmann S Deutsch T Schrimpf A. The impact of the COVID-19 pandemic on avoidance of health care, symptom severity, and mental well-being in patients with coronary artery disease. Front Med (Lausanne). 2021;8:760265. 34977066 10.3389/fmed.2021.760265 PMC8714893

[54] Norris ZA Sissman E O'Connell BK Mottole NA Patel H Balouch E Ashayeri K Maglaras C Protopsaltis TS Buckland AJ Fischer CR. COVID-19 pandemic and elective spinal surgery cancelations - what happens to the patients? Spine J. 2021;21(12):2003-9. 34339887 10.1016/j.spinee.2021.07.019 PMC8321964

[55] Suárez-Huerta ML Gomez-Rice A Carvajal Alvarez M Vazquez Vecilla IC Izquierdo-Nuñez E Fernandez-Gonzalez M Zuñiga-Gómez L Betegon-Nicolas J Sanchez-Campos S. Effect of COVID-19 on quality of life of persons aged >70 years with adult spinal deformity: a cross-sectional case-control study. Medicine (Baltimore). 2022;101(33):e29954. 35984207 10.1097/MD.0000000000029954 PMC9387660

[56] Levinson W Gorawara-Bhat R Lamb J. A study of patient clues and physician responses in primary care and surgical settings. Jama. 2000;284(8):1021-7. 10944650 10.1001/jama.284.8.1021

[57] Best MJ McFarland EG Thakkar SC Srikumaran U. Racial disparities in the use of surgical procedures in the US. JAMA Surg. 2021;156(3):274-81. 33439237 10.1001/jamasurg.2020.6257 PMC7807389

